# Using a Human-Centered Design Process to Evaluate and Optimize User Experience of a Website (InPACT at Home) to Promote Youth Physical Activity: Case Study

**DOI:** 10.2196/52496

**Published:** 2024-07-15

**Authors:** Rebecca E Hasson, Michelle Xie, Dhiraj Tadikamalla, Lexie R Beemer

**Affiliations:** 1 School of Kinesiology, University of Michigan, Ann Arbor, MI, United States; 2 School of Information, University of Michigan, Ann Arbor, MI, United States

**Keywords:** web-based interventions, children, adolescents, child, adolescent, youth, user experience, website, websites, implementation science, human-centered design, human-centred design, HCD, web based, home based, interview, heuristics, interviews, heuristic, competitive analysis, video, videos, YouTube, physical activity, exercise, fitness

## Abstract

**Background:**

Web-based physical activity interventions often fail to reach the anticipated public health impact due to insufficient use by the intended audiences.

**Objective:**

The purpose of this study was to use a human-centered design process to optimize the user experience of the Interrupting Prolonged sitting with ACTivity (InPACT) at Home website to promote youth physical activity participation.

**Methods:**

Qualitative interviews were conducted to assess engagement and pain points with the InPACT at Home website. Interview data were used to create affinity maps to identify themes of user responses, conduct a heuristic evaluation according to Nielsen’s usability heuristics framework, and complete a competitive analysis to identify the strengths and weaknesses of competitors who offered similar products.

**Results:**

Key themes from end user interviews included liking the website design, finding the website difficult to navigate, and wanting additional features (eg, library of watched videos). The website usability issues identified were lack of labeling and categorization of exercise videos, hidden necessary actions and options hindering users from decision-making, error-prone conditions, and high cognitive load of the website. Competitive analysis results revealed that YouTube received the highest usability ratings followed by the Just Dance and Presidential Youth Fitness Program websites.

**Conclusions:**

Human-centered design approaches are useful for bringing end users and developers together to optimize user experience and impact public health. Future research is needed to examine the effectiveness of the InPACT at Home website redesign to attract new users and retain current users, with the end goal of increasing youth physical activity engagement.

## Introduction

Physical activity is one of the most efficacious pathways to promote child health, well-being, and academic achievement [1,2], yet most children and adolescents in the United States are classified as inactive. Less than half (42%) of children ages 6-11 years participate in the recommended 60 minutes of daily physical activity, and this percentage declines as children transition into adolescence [3-5]. Children living in low-resource communities report even lower rates of physical activity [6,7], and the recent COVID-19 pandemic exacerbated these disparities [8,9] by contributing to a 17-minute decline in youth physical activity [8-10]. We have an urgent and unmet need to increase youth physical activity engagement to improve child and adolescent health.

The COVID-19 pandemic also brought renewed attention to prioritizing virtual methods of physical activity promotion as families were sheltering in place and children were attending schools online [11]. Web-based interventions have the potential to improve youth physical activity participation because of their extensive reach, high convenience, immediate feedback, diverse delivery formats, anonymity, and use across different contexts [12-14]. Web-based interventions can reach children and adolescents nearly anywhere at any time through desktops, laptops, and mobile devices [15,16]. Because this generation of children and adolescents spend large amounts of time watching or using screens (4‐6 hours per day for children ages 8‐12 years and up to 9 hours for teens) [17], web-based interventions represent a feasible and accessible strategy to promote youth physical activity engagement.

Evidence is lacking, however, for achieving sustainable physical activity behavior change through the internet. A recent review of web-based physical activity interventions highlighted that despite large developments in internet technology and knowledge of how to design and implement web-based physical activity interventions, website quality remains low [18]. These websites also provided limited social support and educational content [18].

Families play a crucial role in shaping a child’s activity levels by providing various forms of social support [19,20]. This support includes encouragement, participating in activities together, and observing a family member’s involvement in physical activities or sports. For instance, Tandon et al [21] found that in predominantly White households, parental support was linked to an extra 12 minutes of moderate-to-vigorous physical activity per day. Similarly, a study by Graham et al [22] in a diverse population showed that parental support, including role modeling, influenced adolescent activity levels.

Our Interrupting Prolonged sitting with ACTivity (InPACT) at Home program, for example, confirmed the importance of parental support. InPACT at Home was a television and web-based intervention designed to help elementary and secondary school students in kindergarten through grade 12 (K-12) stay physically active and maintain healthy lifestyles during the COVID-19 pandemic [23]. Our preliminary research assessing the feasibility of children using InPACT at Home exercise videos demonstrated that parents encouraging their children, reminding them, and establishing schedules and routines significantly facilitated participation in the program (LR Beemer et al, unpublished data, 2024). These findings underscore the pivotal role of parents in promoting virtual home-based physical activity for youth. Consequently, the design of the InPACT at Home website targets parents, where parental engagement leads to parent support and subsequent increased participation by their children in the exercise videos.

Problems associated with attracting, engaging, and retaining participants in web-based interventions have also been observed [24,25]. While the reach of the InPACT at Home program through public television broadcasting averaged 15,000-20,000 daily viewers, there were only 23 registered users on the program website 1 year after the program launch (website data unpublished). These findings illustrated the potential need for enhanced website design quality and the incorporation of end user input to reach the intended audiences and achieve the planned behavior change.

Proper design has become a critical element needed to engage website users. Poorly designed websites may frustrate users and result in a high “bounce rate,” or people visiting the home page without exploring other pages within the site [26]. On the other hand, a well-designed website with high usability has been found to positively influence visitor retention (revisit rates) and engagement behavior [27,28]. A comprehensive analysis of the usability heuristics of the InPACT at Home program website was not conducted before its launch. Human-centered design represents a unique approach for tailoring web platforms to fit end users, narrowing the gap between efficacious interventions and public health impact. This approach places end users (ie, real people) at the center of the development process, enabling website developers to create programs and platforms that are tailored to the intended audiences’ needs. The end users’ wants, pain points, and preferences are prioritized during every phase of the process to enhance engagement and accessibility of the web-based program [29]. Given the problems associated with attracting, engaging, and retaining users to the InPACT at Home program website, the purpose of this study was to use a human-centered design process to evaluate and optimize user experience to promote website engagement and subsequent youth physical activity participation.

## Methods

### InPACT at Home Program

The InPACT at Home program is an evidence-informed family physical activity program that uses high-quality instructor-led physical activity videos to promote exercise in the home [23,30]. The InPACT at Home website is run on a WordPress platform, hosted by the university, and was developed by a professional web design company. The website is published publicly with log-in features to allow both the program developers and end users to track their completed activities and rewards. End users are awarded badges upon completion of the exercise videos. This feature was added based on previous research conducted in classroom settings that demonstrated significant improvements in youth moderate-to-vigorous intensity physical activity engagement when game design elements were added to the program [31]. Rewards have also been identified as facilitators of youth participation in virtual reality exergaming interventions [32].

A “Challenges” page was added to the website to highlight one health theme each month. Finally, to encourage mindfulness after each workout a postworkout survey was added to the website. End users are encouraged to answer the following questions: “In one or two words, please describe how participating in physical activity makes you feel?” and “If you could tell your friends one or two words about why physical activity is important, what would you say?” Previous research has demonstrated that engaging in self-reflection activities after a positive exercise experience can aid in the continuance of the behavior [33].

Physical education teachers, fitness professionals, pediatric exercise physiologists, athletes, and high school students from across the state of Michigan were recruited and hired to develop exercise videos that were developmentally appropriate and could be completed at home with no or minimal equipment. The types of exercises included were aerobic, isometric strength training, motor skills, sports skills, yoga, and mindfulness training. To supplement the exercise videos, physical activity play cards and family engagement tool kits were developed to provide another opportunity for children and families to move and play together. The movement-based play cards included cardio, strength, mindfulness, flexibility, and “with a buddy” activities. School psychologists, regional school health coordinators, and teachers from across the state of Michigan were hired to develop family engagement tool kits that focused on the following topics: resilience, well-being, focus, nutrition, sleep, family team building, family discussion, personal best, health choices, lifelong skills, substance misuse, schedules, and routines. Each module also included a 20-day challenge that incorporated movement activities. All program materials are hosted on the InPACT at Home website [34].

### Recruitment

Parents of children and adolescents in grades K-12 (ages 5‐17 years) were recruited to participate in this study using a variety of methods: registration opt-in for user research on the parent permission form on the program website, advertisements on the university clinical trials website, and sending out email advertisements to current users. Participants were eligible for inclusion in this study if they had a desktop computer and internet access in their homes, were able to answer questions and complete tasks on a computer, and were able to understand English. Participant eligibility was determined by parents answering a screening questionnaire, after which a member of the research team contacted them by email to confirm their eligibility and schedule the interview. Informed consents were obtained before the start of the study via a web-based survey using Qualtrics software (Qualtrics International Inc). If participants did not agree to participate in the study, the survey ended.

### Qualitative Interviews

One-on-one semistructured interviews with parents of child users were conducted to assess engagement and pain points with the InPACT at Home website. Pain points were defined as specific problems faced by current or prospective website users and included any problems the user experienced when engaging with the website [35]. Interviews were conducted by trained research staff using videoconferencing. Four trained research staff members, all fourth-year undergraduate students at the University of Michigan School of Information, conducted the data analysis. Their training involved four years of coursework and real-world experiential learning through internships within the university’s school. The same research staff conducted all aspects of the research study.

Purposive sampling was used to select participants from the pool of participants who responded to the advertisement. The criteria used for selection included accounting for diversity in race, gender, and age of their child. Using a standardized interview schedule, all participants were asked the same interview questions. Interviewers also asked additional unplanned questions to further assess new information introduced by participants. During the interview, interviewers were able to see participants’ computer screens. Interviews ranged from 45 to 60 minutes in duration and were audio recorded and transcribed verbatim using a transcription company. Participants were compensated US $25 for their time.

### Affinity Mapping

An affinity map involves gathering qualitative information about a target population and organizing it into categories. Initially, it is a useful method for compiling extensive information and data about users from various stages of development, such as user testing, surveys, observations, and feedback collection. The goal is to create an affinity diagram, a tool that visualizes the brainstorming process.

Professional user experience teams typically follow a flexible set of instructions, starting with selecting a topic, forming a cross-functional team, gathering facts and ideas, categorizing items, and devising an action plan. Throughout the session, team members collaborate to generate ideas pertinent to the chosen topic, with each brainstorming session yielding potentially different outcomes. An essential principle of affinity mapping is the absence of absolute right or wrong ways to categorize data; different teams may interpret the data differently and create distinct groups of data points based on collective decisions.

Approaching the data with a fresh perspective is advisable, avoiding premature labeling based on past experiences, as each data set is unique. Moreover, there are no rigid rules on how observations should be articulated; the focus is on gathering data in a manner that aligns with the team’s dynamics.

Using a phenomenological approach, a thematic content analysis was used to examine the data and identify themes that elucidate each participant’s experiences with physical activity programming and the InPACT at Home website. Qualitative data from semistructured interviews were organized into an affinity map using Miro software version 3.11.8 (RealTimeBoard, Inc).

The research staff reviewed the qualitative information, jotting down each observation on a movable card (ie, sticky note). The visual aspect of using sticky notes aids the team in physically visualizing connections between key data points, facilitating a literal connection between ideas. Sticky notes also allow for easy rearrangement and modification of groupings throughout the brainstorming process. The raters collaborated in a single room to jot down observations and identify themes, benefiting from collective brainstorming and free exchange of thoughts.

Using a large whiteboard, patterns in the observations were identified and categorized into groups. Each group was named, and a summary statement was provided regarding what was learned about each group. The analysis team also looked for outlier observations to understand instances where individual participant perspectives differed from the main findings, thus allowing for multiple perspectives and mitigating bias. Regular research meetings were held throughout the data analysis process with research team members possessing qualitative expertise to discuss the progress.

### Heuristic Evaluation

A heuristic evaluation serves to systematically review the current state of a product, identifying usability and experience issues [36,37]. This evaluation is conducted based on Jakob Nielsen’s 10 usability heuristics, which are high-level guidelines grounded in an understanding of human behavior, psychology, and information processing. These principles cover various aspects such as system status visibility, matching with the real world, user control, consistency, error prevention, recognition over recall, flexibility, minimalist design, error recovery assistance, and the provision of help and documentation [38]. These heuristics can be grouped into four main quality components: learnability, efficiency, memorability, and error management.

The term “heuristic” refers to a rule of thumb, and this process is particularly valuable in the early stages of a project due to its cost-effectiveness in analyzing the product being worked on. While it does not replace user research, it aids in identifying and defining the problems within a product. For instance, during evaluations of the InPACT at Home website using Figma software version 3.30 (Figma, Inc), usability issues were identified through the Nielsen process. These issues, such as dead links leading to a blank screen, were detected during internal product evaluations.

All issues identified during evaluations are based on team member observations, while the affinity map consolidates key data points from various sources collected before the evaluation. These data points, derived from user surveys and interviews, represent insights from the target users. Initially, all identified issues are assessed for severity to prioritize them effectively.

Each evaluator assigned a severity rating to usability issues on a scale of 0 (ie, no issue) to 4 (ie, usability catastrophe), accompanied by documentation of the specific violation and recommendations for fixing the problem. These ratings reflect a consensus reached by the group of evaluators, and they help guide decision-making regarding issue resolution.

### Competitive Analysis

The purpose of conducting a competitive analysis is to gain strategic insights into how your product compares to the design solutions offered by competitors. This analysis covers various aspects such as functions, features, user flows, and the emotional response elicited by competitors’ products. The goal is to strategically design your product to outperform the competition. Typically, this analysis is conducted initially to understand how you want your new product to differentiate itself. However, it is beneficial to approach this process iteratively, as competitors are constantly evolving. The key is to draw inspiration from competitors’ solutions and determine what aligns best with your product and its intended users.

We specifically selected the Presidential Youth Fitness Program, YouTube, and Just Dance because we believe their features closely align with those of InPACT at Home. Presidential Youth Fitness Program offers a youth fitness training program aimed at promoting health-related fitness and providing quality resources for physical education, which aligns well with InPACT at Home’s goals of engaging families and promoting physical activity. Similarly, YouTube offers a vast array of functionalities, including promoting healthy lifestyles, and its user-friendly video experience and large user base make it a strong competitor for analysis. Just Dance targets a younger audience and encourages active engagement through video platforms, aligning with InPACT at Home’s objectives. Therefore, we identified these three competitors for comparison based on their alignment with InPACT at Home’s goals and features.

The research team conducted a competitive analysis using Figma software to identify the strengths and weaknesses of InPACT at Home’s competitors offering similar online products promoting physical activities. Research staff analyzed both direct and indirect competitors to identify gaps or opportunities that could give InPACT at Home an edge over its competitors [39]. Five aspects of each website were compared: target audience, first impressions, interactions, visual design, and content, chosen based on their relevance to InPACT at Home’s goals.

Each aspect was rated as “Outstanding,” “Good,” “Okay,” or “Needs work” based on the observed pros and cons. An example of a con for first impressions would be “too many features and complicated user flow,” while a pro would be a “clean, minimalist design.” Our approach involved individual reporting followed by consolidation to generate comprehensive insights based on key takeaways. Information from the competitive analysis was not compared against the InPACT at Home website for benchmarking but instead used as inspiration to determine what aligns best with our website and intended users.

### Ethical Considerations

This study was approved by the institutional review board of the University of Michigan (HUM00192745).

## Results

### Overview

Of the 98 eligible participants who responded to study advertisements, seven parents of children in grades K-12 were contacted and agreed to be interviewed. There were three non-Hispanic White male participants, 1 Asian female participant, two non-Hispanic Black female, and one non-Hispanic White female (average age 41.3, SD 10.2 years). On average, parents had 1.6 (SD 0.8) school-age children (average age 8.4, SD 4.5 years) residing in their household. Of the seven parents, three reported being regularly physically active, and five parents reported their children participated in regular physical activity.

### Affinity Mapping

Thematic saturation was achieved, and Textbox 1 displays the themes and supporting quotes from participants derived from the qualitative interviews conducted. Participant interview responses were categorized into specific website components and included the following: landing/home page, video, current progress and badges, play cards, and overall experience with the website. Responses were further categorized into “likes,” “dislikes/struggled with,” and “wants” as they related to each component. The following themes emerged from the interview responses. The first theme related to “website likes” included the *website design*. Parents noted that they liked that the website was gamified, colorful, and included pictures. Parents also commented on the variety of exercises and resources available to parents. The second theme related to “website dislikes” included *difficult navigation*. Parents noted that there was too much scrolling on the home page. The reflection/record progress survey was difficult to find, and some parents were unable to find the play cards. Finally, the third theme related to “website wants” included *added features*. Parents suggested adding a progress button, a library of watched videos, and more information about the “Challenge” page.

Textbox 1. **Themes emerging from the end user qualitative interviews.**

**Theme related to website likes: liked website design (eg, gamified, colorful, pictures, exercise variation, resources for parents)**

- “The design is simple and a very colorful website.” (P02 Sarah)
- “More motivated to earn badges.” “Like how it provides resources for parents, not only kids.” (P07 Molly)

**Theme related to website dislikes: difficult navigation (eg, too much scrolling on home page, reflection/record progress survey difficult to find, unable to locate play cards)**

- “Too much information and scrolling on the home and landing page.” (P02 Sarah)
- “Could not find the record your progress survey button.” “Was expecting the survey to pop up immediately after finishing the video.” (P03 Matt)
- “Thinks the reflection survey process was challenging because had to click on the back button to go back to the original page.” (P04 Janice)
- “Did not know what a play card is so it was hard to find, and the search bar did not work on the website.” (P06 Nina)

**Theme related to website wants: added features (eg, progress button, library of watched videos, challenge information)**

- “It would be better if there was a feature that stated my progress to put the individual’s current physical activity progress.” “It would be nice to have a library to show already watched videos.” (P03 Matt)
- “Give more information about what kind of badges and what you can do with the challenges.” (P07 Molly)

### Heuristic Evaluation

Table 1 presents the results of the heuristic evaluation conducted on the InPACT at Home website. For the heuristics of consistency and standards, and helping users recognize, diagnose, and recover from errors, the website received a score of 0, indicating that the evaluators did not perceive these as usability issues. Two other heuristic categories, recognition rather than recall and help and documentation were assigned a score of 2, indicating minor usability problems. For three categories, namely match between the system and the real world, flexibility and efficiency of use, and aesthetic and minimalist design, the website received a score of 3, indicating major usability problems. Finally, the heuristics of visibility of system status, user control and freedom, and error prevention were rated with a severity score of 4, representing usability catastrophes.

**Table 1.** Heuristic evaluation for the Interrupting Prolonged sitting with ACTivity (InPACT) at Home website.

Heuristics	Violation	Recommendation	Severity
Visibility of system status	Does not show the user how much time they must wait before a new page is loaded. When the user clicks on the tab, there’s nothing that indicates that the user has clicked on it or is currently clicking on it.	Having a loading icon that pops up when the user clicks on a tab to go to another page to show the user that the new page is loading. When the user clicks on a tab in the navigation bar, have the tab color change to a different color to show the user that the system knows they’re clicking on the right tab.	4
Match between system and the real world	Require the user to think hard about what the category means and what the language implies. For instance, “Topics, Challenges” are not familiar categories to the user. They’ll be thinking about what topics and challenges the website is referring to.	Replace category names with more familiar categories to the user such as replacing “Topics” with “Explore.”	3
User control and freedom	Users do not have the control to exit the reflection survey after finishing watching a video.	Add a button that allows the user to exit the survey if they do not want to fill it out.	4
Consistency and standards	All pages are consistent.	Nothing to change.	0
Error prevention	The reflection survey leads to a dead screen, so users must click the back button to return to the InPACT at Home website. Users can accidentally click log-in on the register page, leading to users having no actual registration and needing to enter all personal information again.	Include another button that allows the user to go back to the home page or have the original button add a new tab that can be closed. Remove the log-in option on the register page.	4
Recognition rather than recall	Users must remember to scroll down to complete the reflection survey since it is not on a screen once you complete an activity. Users must remember which videos are their favorites for future uses.	Move the reflection survey to within the screen when users finish a video/challenge. Incorporate a favorite section where users can easily see which videos they have enjoyed.	2
Flexibility and efficiency of use	While there is an option for progress recording, the information is not displayed on the screen, hindering users from decision-making. Video titles are not descriptive enough to communicate the video content (eg, “Scott Przystas-Short Video 2”).	Notify the users when they complete the video and guide them for progress recording. Subcategorize videos and make each title distinct to one another.	3
Aesthetic and minimalist design	The profile section is not designed with proper grouping and colors—badge colors do not communicate their meaning.	Include section that explains what each color represents or substitute the colored badges into word tags.	3
Help users recognize, diagnose, and recover from errors	InPACT at Home uses this heuristic by providing error messages when the user tries to log in, and if the log-in is incorrect, there will be a message that says why the log-in is not working.	Nothing to change.	0
Help and documentation	Users must understand that their most recent badges and ranks are on their profile page. Users not only have to navigate back from the reflection survey but also must remember which video was most recently watched.	Incorporate a notification that users have earned a badge or reward right after it was achieved. Either a banner or pop-up notification, so users do not need to remember or navigate anywhere else.	2

### Competitive Audit

Table 2 displays the competitive audit comparing the online physical activity experience of each website. YouTube received the highest ratings of the three competitors with “outstanding” ratings in six of the seven categories (ie, desktop web/game experience, accessibility, user flow, navigation, brand identity, and descriptiveness). Just Dance received the second highest ratings with “outstanding” ratings in five of the seven categories (ie, desktop web/game experience, user flow, navigation, brand identity, and descriptiveness). The Presidential Youth Fitness Program website was the lowest-rated website with “good” ratings in three of the seven categories (ie, navigation, brand identity, and descriptiveness).

**Table 2.** Competitive audit comparing the online physical activity experience of the Presidential Youth Fitness Program (PYFP), YouTube, and Just Dance websites.

	First impressions	Interactions				Visual design	Content	
	Desktop web/game experience	Features	Accessibility	User flow	Navigation	Brand identity	Tone	Descriptiveness
PYFP	Okay (+) Clean design (–) Too many features and complicated user flow	Okay (+) Resource guide for parents and educators (+) Awards store for recognition (–) No progress recorder (–) Not able to log in unless users are part of the organization	Needs work (+) Video speed options (+) Only offers 1 language: English (–) No subtitles or closed captions (–) No color-blind mode	Needs work (–) Overwhelming number of user interface elements and content	Good (+) Clear indication of clickable elements (–) Some unfamiliar navigation patterns	Good (+) Visual design communicates organization ethos (–) Visual design does not always support content intuitively	Professional and informative	Good (+) All key information is present (–) Too descriptive
YouTube	Outstanding (+) Well designed and easy to use (+) Modern minimalistic design	Good (+) Any user can create own videos, comment, like, save, and share. (+) Filtering and recommendations features (+) YouTube Kids, providing content that is age appropriate (–) Not categorized	Outstanding (+) Subtitles and closed captions (+) Screen reader, interaction controls, display settings, audio and on-screen text options (+) Offers 75 different languages for site navigation	Outstanding: (+) Straightforward user flow (+) One click sign-up (+) Easy video selection process due to recommendations	Outstanding: (+) Easy basic navigation (+) Clear indication of clickable elements (+) Understandable link labels	Outstanding (+) Strong brand identity including colors, fonts, style, and imagery (+) Visual design communicates company ethos	Sophisticated and informative	Outstanding (+) All key information is present
Just Dance	Outstanding (+) Well designed and easy to navigate (+) Cheerful theme with vibrant colors	Outstanding (+) Support both single and multiplayer mode (+) Kids mode: dancers of any age can enjoy (+) Multiple levels: easy to hard (+) Leaderboards and forums	Good (+) Game can be paused at all times (+) Subtitles for lyrics of the songs (–) Offers 15 different language options (–) No color-blind mode (–) No assist features or ability to set the game speed	Outstanding (+) Fun and easy to use (+) Display song options in digestible categories	Outstanding (+) Easy basic navigation (–) No link labels—only present as icons	Good (+) Modern and trendy design (+) Visual design communicates company ethos	Engaging, concise, and friendly	Outstanding (+) All key information is present

## Discussion

### Principal Results

Given the problems associated with attracting, engaging, and retaining users to the InPACT at Home program website (15,000-20,000 daily viewers through public television broadcasting vs 23 registered website users) [23], the purpose of this study was to use a human-centered design process to evaluate and optimize the user experience to promote website engagement and subsequent youth physical activity participation. Using qualitative methodologies and evidence-based heuristic evaluation approaches, we conducted a series of assessments to examine end user engagement and pain points as well as completed a competitive analysis to identify the strengths and weaknesses of competitors who offered similar products. Both the affinity maps developed from end user interviews and the heuristic evaluation of the InPACT at Home program website revealed several major problems and usability catastrophes in three of the four Nielsen quality components: *learnability*, *efficiency*, and *errors*. All these issues resulted in low usability (difficult to navigate) of the InPACT at Home program website and likely contributed to the low user retention (registration rates) and engagement behavior previously observed. The competitive analysis identified YouTube as the highest-rated competitor with “outstanding” ratings and revealed key features that the InPACT at Home program website could benchmark (ie, desktop web/game experience, accessibility, user flow, navigation, brand identity, and descriptiveness). Taken together, these findings suggest the InPACT at Home website needed numerous modifications to enhance usability. Appropriately, the YouTube website interface provided a road map by which we could improve our design interface to fit end user goals and preferences.

### Comparison With Prior Work

Previous research has demonstrated that parent support is an important determinant of child and adolescent physical activity participation. Data from vEngage, a virtual reality exergaming intervention, suggest that while parents would rather their child perform “real-world” physical activity, they believed the key to engagement was through technology and were willing to support their child’s participation in exergaming [40]. Our recent findings from the InPACT at Home program demonstrated that parent support in the form of encouraging their children, reminding them, and establishing schedules and routines significantly facilitated participation in the program (LR Beemer et al, unpublished data, 2024). These findings provide the rationale for why parents were selected as the target audience. Understanding the pain points of parents in using the InPACT at Home website was vitally important to achieving a “trickle-down effect” for child engagement, and accordingly, issues with learnability, efficiency, and error needed to be addressed.

Nielsen’s usability heuristics framework conceptualizes *learnability* as the ease with which users can accomplish basic tasks the first time they encounter the website design [41]. The goal is to design a clear interface that users can quickly learn and understand. Previous research has demonstrated that users can receive more value from a website with high learnability compared to websites with lower learnability [42]. This is due in part to users being able to adopt the learnable interface much quicker and subsequently accomplish their goals in a shorter amount of time using the website. By having an easier time navigating the website, users will also have an overall better experience with the website, which can contribute to a better retention rate and lower bounce rates [43,44]. Best practices for creating a learnable interface include consistency (eg, giving all the web pages a similar look by positioning elements in the same location), feedback (eg, link color changes that tell the user that an element is clickable), using well-known user interface elements (eg, sticking to industry design best practices), familiarity (eg, user can learn the new interface based on previous knowledge), and testimonials (eg, visual storytelling enabling users to learn and remember information).

In this study, the *learnability* of the InPACT at Home website was deemed low as the program website did not provide timely feedback and used unfamiliar concepts, thereby increasing the time needed to learn how to use the website. The website also contained extraneous information that competed with relevant information needed to complete a task, making it difficult for the end user to understand how to use the website. Themes from end user interviews also confirmed that the website was difficult to navigate. Accordingly, substantial attention to creating a learnable user interface on the InPACT at Home program website was needed to optimize user experience and engagement with program resources.

The *efficiency* of the InPACT at Home website was also deemed low, and *errors* were deemed high. Efficiency measures the speed (or quickness) with which a user can accomplish a task once they have become familiarized with the website design [45]. In other words, *efficiency* is the number of keystrokes or clicks it takes a user to complete a task. Like *learnability*, the more efficient an interface design is, the greater value a user can gain from a website as they can complete a task in a shorter amount of time [27,42]. *Errors* on the other hand are software problems that come from a misconfigured website design, making it difficult to complete a task resulting in user frustration [45]. The InPACT at Home program website did not enable users to have control to exit the reflection survey after finishing watching a video; there were no options for progress reporting on the website, and video titles were nondescriptive; all these factors led to website inefficiencies. In addition, some pages on the website led to dead screens, and there were several error-prone conditions on the registration page; these factors contributed to errors on the website.

### Optimization of the InPACT at Home Website

Based on the recommendations provided by the end users and website evaluators, we have made several updates to the InPACT at Home program website. To overcome the barriers related to *learnability*, we have created custom module content that can easily be searched and filtered by topic and automatically archived. Users can select the type of exercise videos they want to engage in as well as select the family engagement tool kit topics they are most interested in. A recent review identified personalization as a key mechanism of web-based interventions that elicited positive changes in physical activity behaviors [14]. To overcome the barriers associated with *efficiency*, we have created a modified log-in process to direct the visitor to their content. Rather than having to scroll through all 132 exercise videos, their personalized profile page now hosts their preferred content, making resources quicker to access. To overcome the barriers with *errors*, we have identified and removed dead screens throughout the website and redesigned the registration page. The removal of these errors and error-prone conditions should reduce user frustration with the website.

To also be responsive to user preferences, we added exercise intensity levels to each video and a brief description of the video content to each video so that children know which activities they will be doing and what equipment is needed. We have also created QR codes for customized workouts to further enhance the personalization of the site. Many of these improvements were modeled after YouTube features identified in the competitive analysis for website design. Our next step in the website optimization process is to conduct additional user testing to confirm these updates are meeting end user needs. We will begin to monitor engagement with the website and program resources.

This study has several important strengths that are worth mentioning. First, we used a common evidence-based heuristic evaluation and competitive analysis to determine the usability of the InPACT at Home website. Second, analyses were conducted by experienced user interface evaluators and researchers with expertise in qualitative interviewing. Finally, our human-centered design approach enabled end users, evaluators, and website developers to come together to evaluate and optimize the user experience to increase website engagement and eventual youth physical activity engagement.

### Limitations

Limitations of this study also warrant attention. First, we acknowledge that our parent sample exhibits some diversity; however, it is essential to consider other characteristics to ensure a truly diverse sample in this context. These include the age range of children from K-12, geographical distribution, levels of digital literacy, income levels, and patterns of technology use. Further testing may be necessary to ensure that the InPACT at Home website adequately caters to the diverse needs of parents and families across the state of Michigan and beyond. Second, there were few existing registered users at the initiation of this analysis; hence, most of the interviews were conducted with parents who were unfamiliar with or not currently using the InPACT at Home website. This could have led to biased responses and recommendations that are only appropriate for first-time users. Third, while 98 eligible participants responded to study advertisements, time and cost constraints limited our ability to conduct more interviews. Nevertheless, we did achieve thematic saturation, and many of the themes identified in the qualitative interviews were confirmed in the heuristic evaluation. Fourth, we used subjective assessments (evaluator ratings) to determine the accessibility and usability of the InPACT at Home program website, thereby increasing the potential for inconsistency in scoring. To overcome this limitation, all four evaluators completed the affinity mapping and heuristic evaluation together; scores reflected a group consensus. The competitive analysis was completed independently (ie, each team member researched one competitor) and then discussed as a group. Finally, the observational nature of the study precluded our ability to directly conduct comparative user testing of the InPACT at Home website along with its competitors. Despite these limitations, our analysis provided valuable information to our website developers from experienced evaluators and end users that enabled us to make substantive changes to the website to improve usability.

### Conclusions

Most children in the United States are classified as inactive because they do not participate in the recommended 60 minutes of physical activity per day [1,2]. Online and web-based interventions have the potential to improve physical activity engagement because of their extensive reach and accessibility across different contexts [14]. Because this generation of children and adolescents is the first to have their entire childhood influenced by the internet and mobile devices [46], web-based interventions may be uniquely positioned to promote sustainable physical activity participation in this age group. Like most other web-based physical activity interventions, the InPACT at Home program website failed to reach its anticipated impact due to insufficient use by the intended audiences. Problems associated with attracting, engaging, and retaining participants in web-based interventions were likely the result of using a website design with low *learnability*, low *efficiency*, and high *errors*. Human-centered design was an evidence-based approach for optimizing the InPACT at Home program website to fit end user goals and preferences. Behavioral interventionists should consider conducting a comprehensive usability heuristic evaluation *before* the website launch to narrow the gap between efficacious interventions and public health impact.

## Abbreviations

InPACT: Interrupting Prolonged sitting with ACTivity
K-12: kindergarten through grade 12
